# Cellular and Developmental Biology of TRPM7 Channel-Kinase: Implicated Roles in Cancer

**DOI:** 10.3390/cells3030751

**Published:** 2014-07-30

**Authors:** Nelson S. Yee, Abid A. Kazi, Rosemary K. Yee

**Affiliations:** 1 Division of Hematology-Oncology, Department of Medicine, Penn State College of Medicine, Program of Experimental Therapeutics, Penn State Hershey Cancer Institute, Penn State Milton S, Hershey Medical Center, Pennsylvania State University, Hershey, PA 17033, USA; E-Mail: aak3@psu.edu; 2 Schreyer Honors College, Pennsylvania State University, University Park, PA 16802, USA; Penn State Harrisburg School of Humanities, Pennsylvania State University, Middletown, PA 17057, USA; E-Mail: rky5018@psu.edu

**Keywords:** calcium, cancer, development, differentiation, ion channels, magnesium, migration, proliferation, survival, TRPM7

## Abstract

The transient receptor potential melastatin-subfamily member 7 (TRPM7) is a ubiquitously expressed cation-permeable ion channel with intrinsic kinase activity that plays important roles in various physiological functions. Biochemical and electrophysiological studies, in combination with molecular analyses of TRPM7, have generated insights into its functions as a cellular sensor and transducer of physicochemical stimuli. Accumulating evidence indicates that TRPM7 channel-kinase is essential for cellular processes, such as proliferation, survival, differentiation, growth, and migration. Experimental studies in model organisms, such as zebrafish, mouse, and frog, have begun to elucidate the pleiotropic roles of TRPM7 during embryonic development from gastrulation to organogenesis. Aberrant expression and/or activity of the TRPM7 channel-kinase have been implicated in human diseases including a variety of cancer. Studying the functional roles of TRPM7 and the underlying mechanisms in normal cells and developmental processes is expected to help understand how TRPM7 channel-kinase contributes to pathogenesis, such as malignant neoplasia. On the other hand, studies of TRPM7 in diseases, particularly cancer, will help shed new light in the normal functions of TRPM7 under physiological conditions. In this article, we will provide an updated review of the structural features and biological functions of TRPM7, present a summary of current knowledge of its roles in development and cancer, and discuss the potential of TRPM7 as a clinical biomarker and therapeutic target in malignant diseases.

## 1. Introduction

The goal of this article is to review the molecular, cellular, and developmental biology of the transient receptor potential melastatin-subfamily member 7 (TRPM7) channel-kinase, as well as its implicated roles in cancer. Transient receptor potential (TRP) is a superfamily of multiple genes that encode biological membrane proteins, and they function as channels that control passage of various ions across membranes. TRP channels represent a most diverse family within the ion channel superfamily, with a wide range of sequence homology among its members. The structural features, expression, and function of TRP ion channels have been reviewed [1,2,3,4,5,6]. In essence, the TRP proteins share a few known common features including intracellular amino and carboxyl termini, six transmembrane segments with the peptide between segments 5 and 6 forming the channel pore, the TRP domain (a region of 23 to 25 amino acids highly conserved among all TRP members), and their channel pores are permeable to cations only.

TRP ion channels are widely expressed in both electrically excitable and non-excitable cells, and they are mostly localized in the plasma membrane. Activation of TRP channels can be triggered by ligand binding, changes in voltage or temperature, or covalent modification of amino acid residues. As a consequence of channel activation, cellular membrane becomes depolarized, and voltage-dependent ion channels permit transmembrane flow of cations such as Ca^2+^ and Mg^2+^ as well as modulation of the associated signaling pathways. These events form the basis of the functions of TRP channels as cellular sensors and transducers of various physical and chemical stimuli. By detecting changes in temperature, pH, osmolarity, pressure, and ionic concentration, TRP channels mediate a variety of physiological responses. These include perception of a wide range of sensations (light, heat, coolness, touch, force, pain, and taste), ionic homeostasis, muscular contraction, vasomotor control, and others.Through regulation of the intracellular concentration of the cations and the associated signaling pathways, the TRP channels can modulate fundamental cellular processes including cell division, growth, survival, differentiation, and migration.

The TRP family in vertebrates is comprised of eight sub-families; each TRP sub-family shares common architectural features and also possesses unique structural motifs [5]. Based on the protein sequence, homology among members of a subfamily in the same species is about 35% [5]. The TRP melastatin-like (TRPM) sub-family consists of eight members (TRPM1 to TRPM8), and their molecular, biophysical, and functional features have been reviewed [7]. Besides the typical structural characteristics of TRP channels, the amino terminus of each TRPM member contains four melastatin domains while the carboxyl termini vary in length and structure. A coiled-coil region (CCR) is present in all TRPM members and involved in multimerization of the channels. The enzymatic domains of three members (TRPM2, TRPM6, and TRPM7) are distinct structures that are downstream of the CCR. The TRPM channels are differentially selective for cations—TRPM2 and TRPM8 are Ca^2+^-permeable non-selective cationic pores. TRPM6 and TRPM7 are believed to primarily conduct Ca^2+^ and Mg^2+^, TRPM4 and TRPM5 are impermeable to divalent cations but they can influence Ca^2+^ entry through other channels by modulating the membrane potential [8].

Among the TRPM channels, the member TRPM7 has been extensively studied and shown to have growing importance in cellular and developmental biology, as well as in human diseases. The biochemical properties of TRPM7 have been characterized *in vitro*, and the functional roles of TRPM7 have been elucidated in cultured cells and model organisms. In this article, we will provide an overview of the structure-function relationship of TRPM7 channel-kinase. Next, an updated summary of the normal biological functions of TRPM7 and its roles in embryonic development will be presented. We will then review the emerging roles of TRPM7 in various types of cancer, and finally discuss its potential as a tumor biomarker and therapeutic target for prevention, early detection, and personalized treatment of malignant diseases.

## 2. Structure and Functions of TRPM7 Channel-Kinase

TRPM7 is a selective cation permeable channel with protein serine/threonine kinase activity [9,10,11]. TRPM7 channel-kinase is ubiquitously expressed, in contrast to the relatively tissue-specific expression of the other seven members of the TRPM subfamily [12,13,14]. A number of studies have indicated that TRPM7 channel-kinase acts as a cellular sensor and transducer. It regulates ionic homeostasis and modulates cellular responses and physiological functions. Studying the structure of TRPM7 channel-kinase and the relationship with its biochemical and electrophysiological functions is important for understanding the mechanisms underlying its cellular and developmental biology, as well as its roles in human diseases, particularly cancer.

### 2.1. Structure of TRPM7

**Figure 1:**
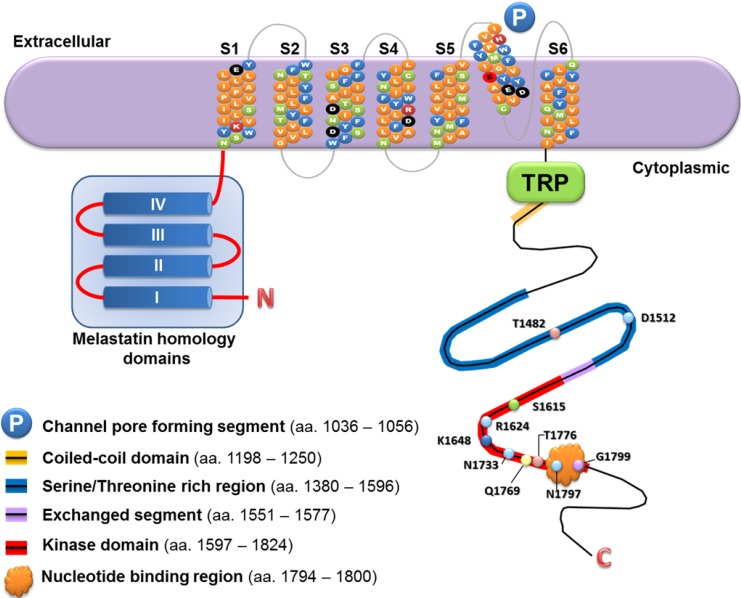
A schematic diagram to illustrate the protein structure of TRPM7 channel-kinase.

The human *TRPM7* gene is located on the long arm of chromosome 15, and it consists of 39 exons that span over 134.34 kb. There are nine splice variants of this gene and only four of the nine transcripts encode protein. The full-length transcript of *TRPM7* contains 7263 nucleotides. The TRPM7 protein is composed of 1,865 amino acids with a molecular weight of 210 kDa. The basic structural features of the TRPM7 protein are homologous and to some extent, conserved among various members of the TRPM channels, as previously reviewed [15]. TRPM7 is similar to TRPM6 (with about 50% identity of protein) and, to a lesser extent, TRPM2, and these are the only known examples of “chanzymes”. Both TRPM7 and TRPM6 channels possess an atypical α-type serine/threonine protein kinase domain in the carboxyl terminus [15]. The channel pore forming segment, serine/threonine rich region, and kinase domain constitute the core components for the functions of TRPM7 (Figure 1). Moreover, the TRPM7 genes and the core functional domains of TRPM7 protein are highly conserved among various species of vertebrates including human, mouse, rat, and zebrafish (NCBI HomoloGene database).

The TRPM7 protein consists of six transmembrane segments (S1 to S6), each about 21 amino acids in length that corresponds to the transverse length of plasma membrane. The amino (N) and carboxyl (C) terminal portions of TRPM7 are shown embracing the transmembrane segments. The channel pore (P) is formed between S5 and S6 and shown slightly off plane. There are 3 negatively charged amino acids (E, E, and D) in the pore forming segment, and they are important for channel function and regulating conductance of cations. Selected amino acid residues including E1047 (in red color within the pore) and those distal to the TRP domain are indicated. Those amino acids have been shown to play important roles in the functions of TRPM7 channel and kinase either by site-directed mutagenesis or as a naturally occurring variant as described in Table 1. The amino acid (aa.) numbers correspond to the major components of human TRPM7 are listed.

The functional TRPM7 channel is a homo-tetramer formed by four TRPM7 monomers assembled in a specific structural conformation presumably by protein interaction through the coiled-coil domain that is highly conserved among the TRPM channels. This prediction is supported by the study showing that the coiled-coils of TRPM8 are required for subunit interactions and self-assembly of the functional tetrameric channels at the plasma membrane. X-ray crystallography and biochemical analysis have revealed the anti-parallel architecture of the coiled-coils of TRPM7, and it is predicted to be important for the specificity of channel assembly [16]. Structure-based comparison of the TRPM members shows that the coiled-coil domain of TRPM6 is most similar to that of TRPM7, and this is consistent with the observations that TRPM7/TRPM6 hetero-tetramers can be formed [17,18,19].

The α-type serine/threonine protein kinase domain of TRPM7 has been shown to form a dimer that can autophosphorylate as well as phosphorylate protein substrates. Based on the crystal structure of TRPM7, two α-kinase domains can assemble into a homo-dimer through interaction of the exchanged segment (aa. 1551 to 1577) that is located proximal to the kinase domain (Figure 1) [20]. This is supported by the *in vitro* experiment showing that the residues 1548 to 1576 of TRPM7 are essential for monomers interaction and kinase activity [21]. In addition, results of site-directed mutagenesis of TRPM7 indicate that the residues 1553 to 1562 are essential for kinase activity, and the residues 1563 to 1570 are critical for assembly of a dimer [21]. Interestingly, the exchanged segment of TRPM6 (residues 1711 to 1740) can substitute that of TRPM7, suggesting that functional TRPM7/TRPM6 kinase hetero-dimers can be formed [21].

### 2.2. Biochemical and Electrophysiological Functions of TRPM7

TRPM7 plays an important role in intracellular Mg^2+^, Ca^2+^, and Zn^2+^ homeostasis. The TRPM7 channel preferentially permits the flow of Mg^2+^ and to a lesser extent Ca^2+^, as well as other physiologically essential divalent metal cations including Zn^2+^, Mn^2+^, Co^2+^. Non-essential and often environmentally toxic metals such as Ni^2+^, Cd^2+^, Ba^2+^, and Sr^2+^ are also permeable through the TRPM7 channel [9,11,22]. In certain types of cells, Mg^2+^ influx through the TRPM7 channel can lead to altered intracellular levels of Ca^2+^ [11,23,24]. Besides the various divalent cations, the TRPM7 channel is also permeable to monovalent ions particularly H^+^. At physiological pH (7.4), the monovalent cationic currents are inhibited; but at acidic pH, the binding affinity of TRPM7 for Mg^2+^ and Ca^2+^ is reduced, and conductance of monovalent cations through TRPM7 becomes permissible [25]. Consistent with these observations, TRPM7 channel exhibits inward proton conductance that can be inhibited by extracellular Mg^2+^ or Ca^2+^ [26]. These studies suggest that these protons may compete with Mg^2+^ and Ca^2+^ for binding sites in the channel pore of TRPM7.

Besides, both TRPM7 and TRPM6 channels play a key role in epithelial reabsorption of Mg^2+^ and regulating total body homeostasis of Mg^2+^ [9,11,27,28,29]. TRPM7 and TRPM6 are constitutively active ion channels permeable to divalent cations, including Mg^2+^. Hetero-tetramers of TRPM7 and TRPM6 can be formed [17,18,19], but the subunit stoichiometry remains to be determined. Reports of single channel conductance and biophysical properties suggest that TRPM7 and TRPM6/7 channels exhibit distinct divalent cation permeability, pH sensitivity, and conductance [19]. Because of the relatively restricted expression of TRPM6 in the kidney and intestine [27,30], in most cases the relevant physiological channel is most likely a TRPM7 homo-tetramer.

Electrophysiological studies indicate that the TRPM7-mediated small inward currents at negative potentials are conducted by divalent ions such as Mg^2+^ and Ca^2+^ whereas the large outward currents mediated by TRPM7 at positive potentials involve monovalent ions such as K^+^ [9,10,11,22]. In resting cells, the channel activity of TRPM7 is suppressed by physiological levels of Mg^2+^ and Mg·ATP [9,31]. The basal constitutive activity of TRPM7 channel is maintained by steady state levels of phosphatidylinositol 4,5-bis-phosphate (PIP_2_) near the channel [32]. The TRPM7 channel can be activated by cyclic adenosine monophosphate (cAMP), by depletion of intracellular free Mg^2+^ or Mg·ATP, and also by intracellular alkalinization [9,25,31,32,33,34]. On the other hand, TRPM7 channel activity can be suppressed by depletion of PIP_2_ (by activation of phospholipase C), high [Mg·ATP]_ic_, high [free Mg^2+^]_ic_ (IC_50_ of 0.6 mM), cytosolic acidity (IC_50_ of pH 6.3), or polyamines such as spermine [9,32,34,35]. The inhibitory effect of intracellular Mg^2+^ can be synergized by halides (Cl^−^, Br^−^, I^−^), and this involves the ATP-binding site in the kinase domain of TRPM7 [36]. Using an aequorin bioluminescence-based assay, organic small-molecule activators of TRPM7 channels have been identified, and they may be useful for probing the mechanism that mediates its functional roles [37]. Taken together, these studies support an important role of TRPM7 channel as a cellular sensor of various physical and chemical changes in the cytosol and in the microenvironment.

TRPM7 is not only an ion channel, it is also a member of an atypical protein kinase family called α-kinases [16]. The TRPM7 kinase can autophosphorylate its serine (predominantly) and threonine residues [38]. By *in vitro* kinase assay using a construct that contains the cytosolic carboxyl-terminal portion of TRPM7, the majority of the autophosphorylation sites were mapped to the Ser/Thr-rich region [39]. Using mass spectrometric proteomic techniques, some of these sites were detected in the intact TRPM7 protein purified from mammalian cells [40]. Mutagenesis and functional studies indicate that autophosphorylation of TRPM7 facilitates phosphorylation of its substrates, while the catalytic activity of TRPM7 kinase remains unaffected [39]. Moreover, TRPM7 can phosphorylate annexin 1 and the heavy chain of myosin IIA, suggesting a role of TRPM7 in vesicle fusion, actomyosin contractility, and cell adhesion [41,42,43]. It has been shown that TRPM7 kinase phosphorylates the serine/threonine sites in the C2-domain of phospholipase γ2 (PLCγ2), suggesting the sensitivity of TRPM7 channels to Mg^2+^ may be regulated through phosphorylation of serine/threonine in PLCγ2 [44]. Besides, TRPM7 regulates Mg^2+^-dependent phosphorylation of the translational factor eEF2 through eEF2-kinase [45]. The kinase activity of TRPM7 is positively regulated by Mg·ATP [31,38,46,47], but reduced at either acidic pH of 4.0 or alkaline pH of 8.4 or 9.0 [34]. These data provide support for the important roles of the kinase in mediating the various functions of TRPM7.

A functional relationship between the kinase activity of TRPM7 and its channel function has been revealed by site-directed mutational analysis in combination with electrophysiological studies. Kinase-inactivating mutations of residues in the kinase domain involved in binding ATP or Zn^2+^ diminished TRPM7-mediated currents [10]. These data suggest that kinase activity of TRPM7 is required for its channel function. On the other hand, mutations of two autophosphorylation sites or a key catalytic site rendered the kinase inactive but did not affect TRPM7-mediated currents or Ca^2+^ influx [38]. These results argue against an essential role of kinase activity in channel function. However, the kinase activity of TRPM7 has been shown to play a modulatory role in channel sensitivity. Point-mutants TRPM7 with deficient phosphotransferase activity exhibited decreased [Mg^2+^]_ic_- or [Mg·ATP]_ic_-induced channel suppression but full channel activation in response to low [free Mg^2+^]_ic_ [11,47]. Moreover, cAMP-mediated enhancement of channel activity and GTPγS-mediated inhibition of channel activity was abolished in cells expressing phosphotransferase-deficient TRPM7 [31]. Results of these studies indicate that the channel function and kinase activity of TRPM7 are functionally coupled.

Furthermore, studies by deletion of kinase domain in TRPM7 (TRPM7^ΔKD^) have demonstrated its regulatory role in channel function. This is supported by the evidence that TRPM7^ΔKD^ exhibited enhanced suppression of channel activity by [Mg^2+^]_ic_ or [Mg·ATP]_ic_ [11], and also abrogated cAMP-mediated up-regulation of channel activity [31]. One study showed that deletion of kinase domain of TRPM7 deprived most of its channel activity [38]. However, studies of endogenously expressed TRPM7 have generated important insights into the role of its kinase and the relationship with channel function. In T-lymphocytes, Fas-receptor-induced apoptosis involves caspase-dependent cleavage of TRPM7 at Asp-1510, rendering the TRPM7 channel with enhanced activity and the pore being dissociated from the kinase domain [48]. A recent report shows that, in normal tissues and cell lines, TRPM7’s kinase is proteolytically cleaved from its channel domain [49]. The released kinase then binds to transcription factors that contain Zn^2+^-binding domain and modifies chromatin through histone phosphorylation, whereas the remaining channel domain gets eliminated [49]. These results suggest that TRPM7’s channel controls cellular influx of Zn^2+^, which is in turn required for its chromatin-modifying kinase to regulate its target genes. These data provide further support for a functional link between the channel and kinase of TRPM7.

Taken together, TRPM7 plays an important role in cellular cationic homeostasis that involves the activities of both channel and kinase. Experimental evidence from various studies supports a reciprocal functional relationship between the channel function of TRPM7 and its kinase domain/activity. On one hand, the cations, particularly Mg^2+^, Ca^2+^, H^+^, and Zn^2+^, conducted by TRPM7’s channel regulate its kinase activity and function. On the other hand, TRPM7’s kinase in turn modulates its channel function in response to [Mg^2+^]_ic_, [Mg·ATP]_ic_ and receptor-mediated signaling that involves cAMP and GTPγS. However, the significance of the channel and kinase activities in the biological functions of TRPM7 likely depends on the particular cell type and the molecular context.

### 2.3. Molecular Determinants of the Functions of TRPM7 Channel-Kinase

*In vitro* analyses of TRPM7 channel-kinase have revealed the molecular features that are important for its physiological functions. Electrophysiological and biochemical studies of site-directed mutagenized TRPM7 and its natural variant have generated insights into the molecular basis underlying the channel’s permeability to Mg^2+^, Ca^2+^, and H^+^, as well as its sensitivity to changes in pH. In addition, the amino acid residues of the TRPM7 protein that influence its kinase activity have been identified. The sites of autophosphorylation and substrate binding, and their functional significance, have been determined (Table 1).

The molecular features responsible for TRPM7 channel’s permeability to Mg^2+^ and Ca^2+^ have been studied by substituting the negatively charged amino acids in the putative pore-forming region (residues 1036–1056, Figure 1). The Glu-1047 is essential for its channel’s conductivity of Mg^2+^ and Ca^2+^, since replacing glutamate by the neutral amino acids, either glutamine or alanine, rendered the TRPM7 channel non-functional in conducting these ions [26,50]. The diminished permeability of TRPM7 channel with E1047Q or E1047A mutation to Mg^2+^ and Ca^2+^ is indicated by decreased binding affinity of these divalent cations, and largely reduced Mg^2+^ and Ca^2+^ currents. Results from similar experiments showed that Glu-1052 also play a contributory role to the channel’s permeability to Mg^2+^ and Ca^2+^ (Table 1). These studies suggest that Glu-1047 and Glu-1052 provide the binding sites for Mg^2+^ and Ca^2+^, and they facilitate the conduction of these divalent cations.

TRPM7 has been shown to possess conductivity to protons, and the negatively charged amino acid residues within the channel pore important for proton conductance have been identified (Table 1). Mutation of Glu-1047 in the putative pore-forming region to glutamine resulted in the TRPM7 channel selective monovalent ions, and the potentiating ability of protons for inward currents disappeared [26,50]. Conversion of Asp-1054 to alanine abolished conductance of protons by TRPM7 channel [26], suggesting a crucial role of Asp-1054 in proton conductivity of TRPM7. Other negatively charged amino acids in the pore region including Glu-1052 and Asp-1059 also facilitate conduction of protons. This is suggested by the study showing that replacement of Glu-1052 or Asp-1059 by alanine dampened the channel’s conductance of H^+^ [26]. Thus, Glu-1052, Asp-1054, and Asp-1059 provide the binding sites for protons, and these results are important for understanding the role of TRPM7 in pathophysiological conditions associated with acidic pH, such as inflammation, ischemia, and cancer.

A number of amino acids located in the kinase domain of TRPM7 (residues 1597-1824, Figure 1) have been shown to influence its kinase activity (Table 1). In particular, Lys-1648 and Gly-1799 are necessary for the phosphotransferase activity and thus crucial for the catalytic function of TRPM7 kinase [11]. Moreover, Arg-1622, Asn-1731, and Thr-1774 contribute to the kinase activity of TRPM7 by providing the binding sites for the phosphate group of ATP [38]. Additionally, Gln-1769 and Asp-1775 are also important for the kinase activity of TRPM7 by providing the sites for metal binding, while Asn-1795 acts as a site for binding of peptide substrate [38]. Mutational analysis of Lys-1648 and Gly-1799 provides insights into the relationship between the kinase activity of TRPM7 and its channel activity. This is demonstrated by the evidence that, abolition of the phosphotransferase and, thus, kinase activities by either K1648R or G1799D mutation leads to decreased inhibition of channel activity by intracellular free Mg^2+^ or Mg·ATP [11].

The serine/threonine rich region (residues 1380–1596, Figure 1) contains the majority of the autophosphorylation sites, and it plays modulatory roles in the channel and kinase activities of TRPM7 (Table 1). This region is important for TRPM7-mediated substrates phosphorylation, which was impaired when the Ser/Thr-rich domain in TRPM7 had been deleted [39]. The evolutionarily conserved Thr-1482 has been shown to be a site for autophosphorylation. The TRPM7 variant T1482I was identified in some patients with neurodegenerative diseases that are associated with prolonged exposure to an environment deficient in Ca^2+^ and Mg^2+^ [51]. T1482I is a missense mutation, and functional studies by mutagenesis of Thr-1482 to isoleucine demonstrated increased sensitivity of the TRPM7 channel to inhibition by intracellular Mg^2+^ without affecting the kinase activity [51]. On the other hand, Ser-1511 and Ser-1567 were identified as the major sites of autophosphorylation of TRPM7. However, changing either or both of these serine residues to alanine did not affect the channel activity or its sensitivity to intracellular Mg^2+^-mediated inhibition [38,51]. Moreover, mutations of Tyr-1553 and Arg-1558 caused reduction of the kinase activity of TRPM7 [21]. These studies indicate that Thr-1482, Tyr-1553, and Arg-1558 provide the sites for regulation of the channel and kinase activities of TRPM7.

In addition to Lys-1648, Gln-1769, Asp-1775, and Gly-1799 as described above, a number of amino acid residues that represent the binding sites for nucleotide and metals have been predicted [52]. The amino acids Arg-1624, Glu-1720, and Asp-1777 are predicted as the sites of nucleotide (such as ATP) binding, whereas the amino acids His-1753, His-1810, Cys-1812, and Cys-1816 as the sites of metal (such as Zn^2+^) binding. These predicted binding sites of nucleotide and metal are all located within the kinase domain (aa. 1597–1824, Figure 1). It has been shown that TRPM7-mediated currents are positively regulated by intracellular Mg·ATP and Mg·GTP [9] and cAMP [31]. Functional studies of these nucleotide and metal binding sites will be valuable for gaining insights into the mechanistic role of TRPM7 in sensing the metabolic state of the cells.

**Table 1:** Molecular determinants of the functions of TRPM7 channel-kinase. The amino acids and the associated functions as determined by site-directed mutagenesis of TRPM7.aa, amino acids; h, human; m, mouse.

Mutations of Amino Acids	Effect on TRPM7 Function	Reference
**Channel Pore Forming Segment (Human, aa. 1036–1056)**		
**m E1047Q** (Glu→Gln) or **h E1047A** (Glu→Ala)	Loss of channel permeability to Mg^2+^ and Ca^2+^.	[50,53]
**m E1052Q** (Glu→Gln)	Decreased Mg^2+^ and Ca^2+^ binding, and reduced Mg^2+^ and Ca^2+^ currents.	[50]
**h E1052A** (Glu→Ala)	Decreased Mg^2+^ and Ca^2+^ binding, and reduced Mg^2+^ and Ca^2+^ currents. Partial reduction of proton conductivity.	[26,53]
**h D1054A** (Asp→Ala)	Loss of proton conductivity.	[26]
**h D1059A** (Asp→Ala)	Partial reduction of proton conductivity.	[26]
**Serine/Threonine Rich Region (Human, aa. 1380–1596)**		
**h T1482I** (Thr→Ile) (a natural variant) (autophosphorylation site)	Increased sensitivity of channel to Mg^2+^-mediated suppression, and decreased current even at reduced [Mg^2+^].	[51,54]
**m D1510** (Asp)	Caspase-mediated cleavage at Asp-1510 resulted in up-regulated channel activity.	[48]
**m S1511A** (Ser→Ala) (autophosphorylation site)	No change in Ca^2+^ influx or sensitivity to Mg^2+^-mediated inhibition.	[38]
**m S1567A** (Ser→Ala) (autophosphorylation site)	No change in Ca^2+^ influx or sensitivity to Mg^2+^-mediated inhibition.	[38]
**m Y1553F** (Tyr→Phe)	~75% of wild-type kinase activity.	[21]
**m Y1553L** (Tyr→Leu)	~50% of wild-type kinase activity.	
**m Y1553A** (Tyr→Ala)	~35% of wild-type kinase activity.	
**m R1558A** (Arg→Ala)	~15% of wild-type kinase activity.	[21]
**Kinase Domain (Human, aa. 1597–1824)**		
**m R1622L** (Arg→Leu) (binding of PO_4_^3−^ of ATP)	<1% of wild-type kinase activity.	[38]
**h K1648R** (Lys→Arg) or (phosphotransfer activity)	Diminished kinase activity. No change in channel activation in response to decreased free Mg^2+^ or Mg·ATP. Attenuated suppression of channel activity in response to free Mg^2+^ or Mg·ATP.	[11]
**m K1646R** (Lys→Arg) (phosphotransfer activity)	<1% of wild-type kinase activity.	[38]
**m K1727A** (Lys→Ala)	<1% of wild-type kinase activity.	[38]
**m N1731V** (Asn→Val) (binding of PO_4_^3−^ of ATP)	<1% of wild-type kinase activity.	[38]
**m E1760A** (Glu→Ala)	~15% of wild-type kinase activity.	[21]
**m D1765N** (Asp→Asn) or **m D1765A** (Asp→Ala)	<1% of wild-type kinase activity.	[38]
**m Q1767N** (Gln→Asn) or **m Q1767A** (Gln→Ala) (metal binding)	<1% of wild-type kinase activity.	[38]
**m T1774S** (Thr→Ser)	<1% of wild-type kinase activity.	[38]
**m T1774A** (Thr→Ala) (binding of PO_4_^3−^ of ATP)	~6% of wild-type kinase activity.	
**m D1775A** (Asp→Ala) (metal binding)	<1% of wild-type kinase activity.	[38]
**m N1795A** (Asn→Ala) (binding of peptide substrate)	~2% of wild-type kinase activity.	[38]
**h G1799D** (Gly→Asp) (phosphotransfer activity)	Diminished kinase activity. No change in channel activation in response to decreased free Mg^2+^ or Mg·ATP. Attenuated suppression of channel activity in response to free Mg^2+^ or Mg·ATP.	[11]

The molecular basis for the channel’s permeability to Mg^2+^, Ca^2+^, and H^+^, pH sensitivity, and kinase activity has been increasingly revealed. Knowledge of the molecular determinants is important for understanding the various functions of TRPM7. The relationship between these sites of TRPM7 and its pleiotropic functions, both *in vitro* and *in vivo* remains to be discovered. Continued studies to identify and characterize the molecular determinants of endogenous TRPM7 are expected to provide insights into the mechanisms underlying its cellular functions and physiological responses.

## 3. TRPM7 Channel-Kinase in Normal Cellular Functions and Embryonic Development

TRPM7 channel-kinase plays regulatory roles in diverse biological processes in various types of cells. Besides, genetic studies in model organisms have revealed pleiotropic functions of TRPM7 during embryonic development. Consistent with its ubiquitous expression, TRPM7 channel-kinase serves a functional role in a variety of cell types. However, the molecular basis for the functions of TRPM7 in each type of cells remains to be determined.

### 3.1. TRPM7 in Cellular Processes and Physiological Functions

Studies have indicated that TRPM7 plays important roles in multiple cellular processes including survival, proliferation, differentiation, growth, and migration (Table 2). In most cell types being studied, silencing of TRPM7 expression impairs these biological events, suggesting a crucial requirement of TRPM7. In contrary, TRPM7 contributes to cell death in anoxic neurons and in umbilical vein vascular endothelia, suggesting an anti-survival role of TRPM7 in these cell types. It has been demonstrated that the opposing effects of silencing TRPM7 on growth and migration of human microvascular endothelial cells (HMEC) and human umbilical vein endothelial cells (HUVECs) is partly related to different phosphorylation status of ERK. This evidence illustrates an important aspect that the functional roles of TRPM7 channels vary among cell types, likely dependent on the molecular context.

**Table 2:** Functional roles of TRPM7 channel-kinase in normal cell types.

Cell Type	Functional Roles of TRPM7 Channel-Kinase	References
Lymphocytes	- Required for Mg^2+^-dependent viability and proliferation of chicken B lymphocytes (DT-40). - Required for proliferation involving phosphoinositide 3-kinase. - Required for differentiation. - Required for survival of T lymphocytes by preventing Fas-induced apoptosis.	[9,11,48,55]
Neurons	- Oxidative stress activates TRPM7, which mediates anoxic death in human neurons; suppression of TRPM7 prevents anoxic neuronal death. - Facilitates fusion of cholinergic vesicle with plasma membrane and neurotransmitter release in cholinergic synaptic vesicles.	[23,56,57,58,59]
Interstitial cells of Cajal	- Required for pacemaker activity of mouse duodenum. - Expressed in the interstitial cells of Cajal of human colon and small intestine and involved in the generation of the slow waves.	[60,61]
Melanoblasts	- Required for survival of melanophores in zebrafish larvae.	[62,63]
Vascular smooth muscle cells	- Functional TRPM7 channels translocate to plasma membrane in response to fluid flow. - Angiotensin II promotes proliferation of VSMCs in ascending aorta by increasing TRPM7 protein via Ca^2+^-influx-mediated activation of the Pyk2-ERK1/2-Elk-1 pathway.	[64,65]
Osteoblasts	- Required for platelet-derived growth factor-induced proliferation and migration of human osteoblast MG-63 cells.	[66,67]
Cervical epithelia	- Required for volume regulation as TRPM7-like currents activated by osmotic swelling-induced mechanical stretch of human cervical cancer HeLa cells.	[68]
Mast cells	- Required for survival of human lung mast cells and human mast cell lines (LAD2, HMC-1).	[69]
Fibroblasts	- Membrane tension activates TRPM7 channels and Ca^2+^ flickers, directing migration in human embryonic lung fibroblasts. - Transforming growth factor-β increased expression of TRPM7 in human atrial fibroblasts associated with myofibroblast differentiation and fibrogenesis in atrial fibrillation.	[24,70]
Vascular endothelia	- Silencing TRPM7 promotes growth/proliferation and nitric oxide production viaERK in human umbilical vein vascular endothelial cells (HUVECs). - Silencing TRPM7 inhibits growth and migration of human microvascular endothelial cells (HMEC) but stimulates growth of HUVECs, partly because of impaired phosphorylation of ERK in HMEC. - Inhibition of TRPM7 leads to increased cell growth and migration in HUVECs. - TRPM7 contributes to hyperglycemia-induced injury of HUVECs.	[71,72,73]
Bone marrow derived mesenchymal stem cells	- Required for survival of mouse bone marrow-derived mesenchymal stem cells; expression increased during osteogenesis suggesting its involvement in differentiation.	[74]
Embryonic stem cells	- Kinase domain, but not kinase activity, is required for proliferation of mouse embryonic stem cells.	[29]
Pancreatic epithelia	- Required for proliferation, cell cycle progression, and growth involving Mg^2+^ and Soc3a in exocrine pancreatic epithelia of zebrafish larvae.	[75]
Hepatic stellate cells	- Required for survival by preventing TRAIL-induce apoptosis. - Regulates platelet-derived growth factor-BB-induced proliferation via PI3K and ERK in a rat hepatic stellate cell line (HSC-T6). - Required for activation and proliferation of HSCs by preventing endoplasmic reticulum stress-mediated apoptosis.	[76,77,78]
Atrial myocytes	- TRPM7-like current was recorded in human atrial myocytes, and expression of TRPM7 is up-regulated in atria with atrial fibrillation or membrane rupture.	[79,80]
Kidney cells	- TRPM7 contributes to elevated level of reactive oxygen species that leads to cell rounding mediated by the p38 MAPK/JNK-dependent activation of the Ca^2+^-dependent protease calpain in the immortalized human embryonic kidney cells (HEK 293), and during ischemia reperfusion in the mouse transplanted kidney.	[81,82,83]
Adipocytes	- Required for proliferation and differentiation of 3T3-L1 pre-adipocytes.	[84]
Prostate epithelia	- Increased Ca^2+^ to Mg^2+^ ratio in human prostate epithelia enhances. TRPM7-mediated currents and promotes cellular entry of Ca^2+^, leading to increase in cell proliferation.	[85]

The fundamental roles of TRPM7 in cellular processes correlate with its capability of mediating the normal physiological functions in various organs (Table 2). These include synaptic neurotransmission, intestinal peristalsis, regulation of vascular tone, bone growth, and others. Some of these are consistent with the ability of TRPM7 to sense and transduce signals of mechanical stress from food digestion and absorption, blood flow, skeletal support, cervical stretch, and wound healing. Additionally, TRPM7 is implicated in atria with atrial fibrillation or membrane rupture, ischemia reperfusion in transplanted kidney, and hyperglycemia-induced injury in vascular endothelia. These data suggest that endogenous levels of TRPM7 are involved in the protective mechanisms in response to oxidative and metabolic stress under those pathological conditions. Further studies *in vivo* are expected to help understand how TRPM7 channel-kinase mediates the physiological functions of various organs.

**Figure 2:**
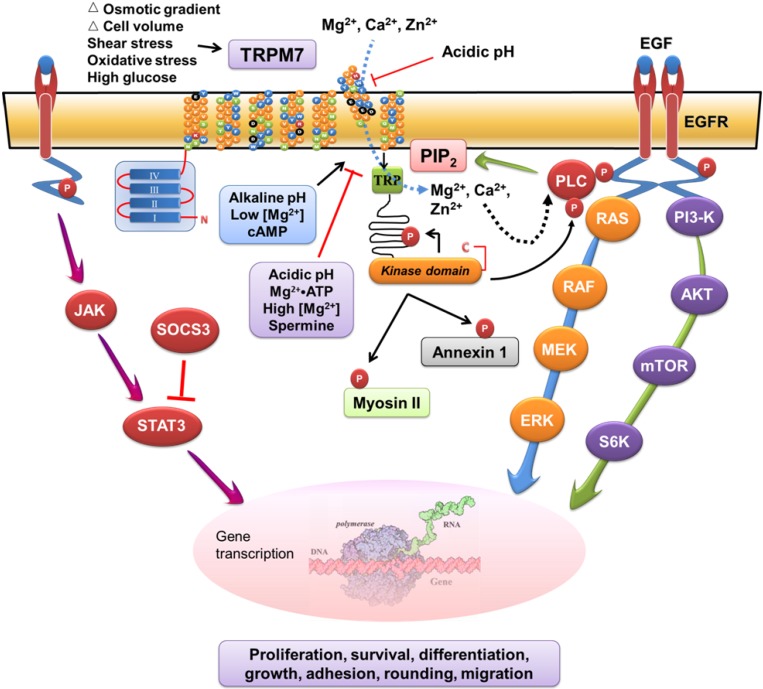
A working model of the signaling mechanisms that mediate the functional roles of TRPM7.

Accumulating evidence has begun to reveal the signaling mechanisms that mediate the cellular functions of TRPM7. Depending on the cell types, the TRPM7-regulated biological functions involve interactions and modulations of the signaling pathways induced by mitogens, stress, and inflammatory cytokines. On the basis of the current evidence, a working model for the signaling mechanism of TRPM7 channel-kinase is proposed (Figure 2). In this model, the TRPM7 channel-kinase acts as a cellular sensor of the physical and chemical stimuli such as mechanical stretch, oxidative stress, changes in cell volume or osmolar gradient, and alterations in extracellular or cytosolic pH. It also acts as a signal transducer by controlling ionic fluxes and modulating the mitogen- and cytokine-induced signaling pathways. However, continued studies are indicated to decipher the signaling mechanisms that mediate the functional roles of TRPM7 in each cell type under various physiological and pathological conditions.

The channel and kinase of TRPM7 are constitutively active in resting cells. In response to various extracellular or cytosolic stress or stimuli, conduction of Mg^2+^ and Ca^2+^ through the TRPM7 channel can be up- or down-regulated. The TRPM7 kinase can phosphorylate itself, as well as cytosolic substrates. The resulting changes in ionic homeostasis and phosphorylation events produce activation or inhibition of the effector molecules in the epidermal growth factor- or other cytokines-induced signaling pathways. These lead to transcription and translation of the cell cycle regulators, senescence-associated genes, and motility mediators. As a consequence, TRPM7 channel-kinase modulates cellular proliferation, survival, differentiation, growth, adhesion, rounding, and migration, which underlie its mediated functions in physiological responses and embryonic development.

In summary, TRPM7 channel-kinase plays diverse functional roles in cellular processes and physiological activities. Thus far, results of those studies indicate that TRPM7 channel-kinase acts as a pivotal connection between the physicochemical stimuli of the cells/microenvironment and the biological responses. The ubiquitous expression of TRPM7 further underscores its essential functions in essentially all cell types. Consistent with its fundamental roles of TRPM7 in various cellular and physiological processes, as well as its requirement for survival and proliferation of stem cells, TRPM7 channels have been shown to play important roles in various aspects of embryonic development.

### 3.2. TRPM7 Channel-Kinase in Early Embryonic Development and Organogenesis

Studies in model organisms have begun to elucidate the developmental roles of TRPM7, and the evidence to date indicates that TRPM7 is important for embryonic viability, gastrulation, and organogenesis (Table 3). Experimental studies by homozygous deletion of *Trpm7* in mouse have indicated that TRPM7 is essential for viability of embryos. In Xenopus, results of the study by selective deletion of the TRPM7 channel suggest that the channel activity of TRPM7 plays crucial roles in gastrulation. Zebrafish embryos and larvae with loss-of-function mutations in *trpm7* are viable and capable of developing organs. Besides, tissue-specific mutation of *Trpm7* in genetically engineered mice enables studying the developmental roles of Trpm7 in certain organs. Thus far, the zebrafish and mouse models have elucidated the pleiotropic roles of TRPM7 in early organogenesis.

**Table 3:** Developmental roles of TRPM7 channel-kinase.

Developmental Processes	Mutant Phenotypes	Functional Roles	References
Embryogenesis	- Early embryonic lethality between E 6.5 and E7.5 in mouse.	- Required for intestinal absorption of Mg^2+^ and whole body magnesium homeostasis.	[29,86]
Gastrulation	- Defects in cell polarization and alignment during convergent extension in Xenopus.	- TRPM7 channel but not the kinase domain required for regulating polarized cell movements during gastrulation involving Mg^2+^ via non-canonical Wnt signaling and modulation of the small GTPase Rac levels.	[87]
Melanogenesis	- Skin hypopigmentation in zebrafish larvae.	- Required for survival of melanophores in zebrafish larvae. - Loss-of-function mutation in Trpm7 leads to cell death of melanophores that is dependent on melanin synthesis.	[62,63,75,88,89,90,91]
Skeletogenesis	- Skeletal deformities in zebrafish with accelerated endochondral ossification and delayed intra-membranous ossification. - Dwarf zebrafish adults.	- Not reported.	[62]
Thymopoiesis	- Selective deletion of *Trpm7* in T-cell lineage accelerates thymic involution in mouse.	- Required for differentiation and maintenance of thymic epithelia. - Required for STAT3 activity in thymic medullary cells.	[86]
Nervous system	- Defects in touch-response in zebrafish larvae. - Paralysis of hind legs of mouse with deletion of *Trpm7* in committed neural crest progenitors; loss of large-diameter sensory neurons in lumbar dorsal root ganglion of mouse embryos depleted of TRPM7.	- Possibly required for synaptic release of neurotransmitters between sensory neurons and interneurons in zebrafish larvae. - Required for development of neural crest progenitors into dorsal root ganglion sensory neurons in mouse. - Required for differentiation or function of dopaminergic neurons in zebrafish larvae.	[59,92,93]
Nephrogenesis	- Nephrolithiasis in zebrafish larvae. - Defect formation of kidney with relatively few glomeruli and large renal cysts in mouse.	- Required for homeostasis of whole body Mg^2+^ and Ca^2+^ in zebrafish involving stanniocalcin 1 and fibroblast growth factor 23.	[92,94]
Exocrine pancreatic organogenesis	- Relatively small pancreas with immature acini and hypomorphic ducts in zebrafish larvae.	- Required for pancreatic epithelial proliferation and growth, which are sensitive to Mg^2+^ in extracellular medium and involving Socs3a.	[75,90,91]

TRPM7 is functionally required for normal development of skin pigment, skeleton, thymus, nervous system, kidney, and exocrine pancreas (Table 3). These data are based on the phenotypes of the zebrafish carrying germ-line mutations in *trpm7* and that of the genetically engineered mice with selective deletion of *Trpm7* in T-lymphocyte lineage. During organogenesis, TRPM7 has been shown to play pleiotropic roles including cellular proliferation, survival, cell cycle progression, growth, and differentiation. Depending on the organ systems involved, TRPM7-mediated control of Mg^2+^ and/or Ca^2+^ homeostasis plays a crucial role. The negative role of Socs3a in Trpm7-regulated exocrine pancreatic development in zebrafish is in agreement with the requirement of STAT3 in TRPM7-regulated differentiation and maintenance of thymic epithelia in mouse. However, the potential roles of TRPM7 in the other organ systems as well as the mechanisms that mediate the functions of TRPM7 in vertebrate organogenesis remain to be discovered. Future developmental studies of TRPM7 are expected to generate new knowledge regarding the biological mechanisms that mediate its functions.Furthermore, studies of TRPM7 channel-kinase in diseases states particularly cancer may help shed new lights on the normal functions of TRPM7 and the underlying mechanism.

## 4. Expression and Roles of TRPM7 in Cancer

### 4.1. Oncologic Roles of TRPM7

Accumulating evidence has implicated contributory roles of TRPM7 channels in a variety of human malignancies (Table 4). It has been demonstrated in pancreatic adenocarcinoma, breast carcinoma, and head/neck cancer, TRPM7 is aberrantly over-expressed in tissue specimens and/or cell lines. Results of studies in culture using cancer cell lines have shown that TRPM7 contributes to cellular proliferation, survival, cell cycle progression, migration, and invasion. These are consistent with the functional roles of TRPM7 in most of the normal cell types and during organogenesis as discussed above (section 3). These results also reflect the importance of developmental studies of TRPM7 for understanding how abnormality in TRPM7 and its signaling mechanism contribute to neoplasia.

**Table 4:** Expression and roles of TRPM7 channels in various human malignancies.

Cancer	Expression	Functional roles of TRPM7	References
Pancreatic adenocarcinoma	- Increased in human pancreatic adenocarcinoma tissues and cell lines. - Increased in chronic pancreatitis, pancreatic intra-epithelial neoplasms	- Required for cellular proliferation and cell cycle progression involving Mg^2+^. - Required for preventing replicative senescence. - Required for cell migration involving Mg^2+^. - Required for cell invasion.	[75,95,96,97,98]
Breast carcinoma	- Over-expression in human breast carcinoma tissues and cell lines. - Increased expression in infiltrating ductal carcinoma with microcalcifications - Somatic mutation T720S (Thr→Ser) in a breast infiltrating ductal carcinoma	- Required for cancer cell proliferation *in vitro*. - Required for cancer cell migration *in vitro* and tumor metastasis in a mouse xenograft model. - Waixenicin A, TRPM7 blocker, inhibits growth and survival of breast cancer cells MCF-7. - TRPM7 involved in estrogen receptor-negative metastatic breast cancer cells migration through kinase domain. - Involved in ginsenoside Rd-induced apoptosis in cells. - Involved in epithelial mesenchymal transition. - TRPM7 mediates migration and invasion of breast cancer cells (MDA-MB-435) involving phosphorylation of Src and MAPK.	[99,100,101,102,103,104,105,106,107]
Gastric carcinoma	- Expressed in human gastric adenocarcinoma cell lines (AGS, MKN-1, MKN-45, SNU-1, SNU-484) - Somatic mutation M830V (Met→Val) in gastric adenocarcinoma	- Required for cell survival involving Mg^2+^. - Waixenicin A, TRPM7 blocker, inhibits growth andsurvival of gastric cancer cells AGS. - Involved in ginsenoside Rd-induced apoptosis AGS cells.	[105,107,108,109,110]
Head and neck Carcinoma	- Expressed in FaDu cells and SCC-25 cells. - High expression in 5-8F cells, low expression in 6-10B cells.	- Required for cell growth and proliferation. - Required for migration of nasopharyngeal carcinomacells (5-8F and 6-10B). - Proliferation of FaDu hypopharyngeal squamous cells (FaDu) inhibited by midazolam that targets TRPM7.	[111,112,113]
Retinoblastoma	- Existence in 5-8F cells	- Required for cell proliferation. - Required for 5-8F cell migration.	[114]
Melanoma	- Expressed in cell lines	- Not reported.	[63,115]
Lung carcinoma	- Expressed in A549 cells	- Required for migration of A549 cells.	[116]
Erythroleukemia	- TRPM7-like currents in cell lines.	- Not reported.	[117]
Colon cancer	-TRPM7 (Thr1482Ile) polymorphism	- TRPM7 (Thr1482Ile) polymorphism associated with elevated risk of both adenomatous and hyperplastic polyps. - Individuals with TRPM7 (Thr1482Ile) polymorphism with a high Ca:Mg ratio intake in diet at a relatively high risk of developing adenoma and hyperplastic polyps.	[54]
Leukemia	- Not reported.	- Waixenicin inhibits and T cell leukemia (Jurkat T lymphocytes) and rat basophilic leukemia cells (RBL1) through blocking TRPM7 channel activity.	[118]
Neuroblastoma	- Not reported.	- In mouse neuroblastoma cells (N1E-115), TRPM7 promotes formation of Ca^2+^ sparking and invadosome by affecting actomyosin contractility independent from Ca^2+^ influx.	[119]
Ovarian carcinoma	- Somatic mutation S406C (Ser→Cys) in ovarian serous carcinoma	- Not reported.	[107]
Prostate cancer	- Expressed in human prostate cancer cell line DU145	- Increased Ca^2+^ to Mg^2+^ ratio in prostate cancer cells enhances TRPM7-mediated currents and promotes cellular entry of Ca^2+^, leading to increase in cell proliferation.	[85]

This important point can be illustrated by TRPM7 in pancreatic cancer. The discovery of the developmental roles of Trpm7 channel-kinase in exocrine pancreas of zebrafish led to identification of its expression and role in human pancreatic adenocarcinoma [75]. TRPM7-mediated cellular proliferation of exocrine pancreatic epithelia in zebrafish larvae and in human pancreatic cancer is Mg^2+^-dependent. This is indicated by the ability of supplementary Mg^2+^ in culture medium to reverse the proliferative defect of pancreatic epithelia in zebrafish with germ-line loss-of-function mutations in *trpm7* [75]. Additionally, down-regulation of *TRPM7* in human pancreatic cancer cells inhibited proliferation by arresting the cells in the G_0_/G_1_ and G_2_/M phases of the cell cycle, and impaired cell migration and invasion; these effects could be reversed by Mg^2+^ supplementation [75,95,97,120]. Moreover, small interfering RNA mediated silencing of TRPM7 induced senescence-associated β-galactosidase in pancreatic adenocarcinoma cells, suggesting a novel role of ion channels in replicative senescence of cancer [98].

These data along with the other studies provide support for important roles of TRPM7, Mg^2+^, and Ca^2+^ in cancer [121]. However, the signaling pathways and the mechanisms that mediate the various cellular effects of TRPM7 in cancer cells remain to be determined. We hypothesize that aberrantly expressed TRPM7 and its regulated homeostasis of Mg^2+^ and Ca^2+^ modulate the epidermal growth factor (EGF)- or other mitogens-induced signaling pathways. These lead to perturbation of the signaling mediators and nuclear events, resulting in uncontrolled proliferation, survival, growth, and invasion of cancer cells (Figure 2).

Thus far, the experimental evidence implicates TRPM7 channel-kinase in multiple hallmarks of cancer, including uncontrolled cell cycle progression, survival, proliferation, migration, invasion, and epithelial-mesenchymal transition. It will be of great interests to determine how the capacity of TRPM7 channel-kinase sensing the physical and chemical changes inside the cells and in the microenvironment contributes to neoplasia. Equally important are the mechanistic roles of TRPM7 channel-kinase in the multistep process of carcinogenesis *in vivo*, such as tumor initiation, growth, invasion, and metastasis.

### 4.2. Potential Role of TRPM7 as a Cancer Biomarker and Therapeutic Target

The evidence showing over-expression of TRPM7 in cancer tissues and cell lines suggests that these ion channels have the potential of being developed into cancer biomarkers for prevention, early detection, prognostication, and predicting/monitoring therapeutic responses. This is supported by the finding that aberrant over-expression of TRPM7 in pancreatic adenocarcinoma positively correlates with tumor size and stage [75,96,97,98,120]. Moreover, the epidemiological finding of the TRPM7 variant T1482I (previously identified in patients with neurodegenerative diseases [51]) in association with dietary intake of Ca^2+^/Mg^2+^ and colonic adenoma/polyps supports further exploration of TRPM7 as a predictive biomarker of cancer [54]. This may help develop strategy for early detection and screening of cancer as well as prevention of cancer through dietary interventions.

Besides, results of multiple studies showing the proliferative and pro-invasion roles of TRPM7 in cancer cells suggest that targeting TRPM7 offers an opportunity of developing new anti-tumor therapies. Chemical inhibitors of TRPM7 channel activity have been shown to produce gastric cancer cells death [104,105,109] and anti-proliferative effects in pancreatic adenocarcinoma cells [122]. The novel finding of RNA interference-mediated silencing of *TRPM7* induces replicative senescence in pancreatic adenocarcinoma cell lines [98] suggests a therapeutic approach complementary to conventional agents that target apoptosis. This is supported by the evidence that small interfering RNA directed against TRPM7 enhances cytotoxicity in pancreatic cancer cells when combined with the chemotherapeutic drug [98]. Results of these studies suggest a potential role of TRPM7 as a therapeutic target that can be exploited by using tumor-specific delivery of anti-cancer agents in malignant diseases.

## 5. Conclusion and Future Perspectives

The TRPM7 channel-kinase is an important cellular sensor and transducer of physical and chemical stress. TRPM7 plays crucial roles in a variety of biological processes, physiological functions, and embryonic development. Progressive advances have been made in deciphering the molecular basis of the activities of the TRPM7 channel and kinase. Increasing evidence has revealed the pleiotropic roles of TRPM7 in cellular and physiological responses in different cell types. Consistent with its cellular functions, TRPM7 is essential in diverse aspects of embryonic development and organogenesis. The aberrant expression of TRPM7 in some malignant neoplasms and its variable requirement for the malignant phenotypes suggest overlapping and unique roles of TRPM7 in cancer arising in each organ.

Future research on TRPM7 channel-kinase is indicated with a focus on several areas: (1) characterize the molecular determinants of the TRPM7-mediated cellular functions and determine their significance *in vivo*; (2) understand how TRPM7 senses and transduces the various physical and chemical changes in the microenvironment and internal milieu of each type of cells; (3) dissect the signaling pathways that mediate the various functions of TRPM7 in each cell type and organ system; (4) define the role of TRPM7 in the multisteps of neoplastic formation and progression, and elucidate the mechanistic roles of TRPM7 in each cancer type; and (5) exploit TRPM7 as a clinical biomarker and therapeutic target for prevention, early detection, and personalized treatment in oncology.
